# Secondary Multiple Intraabdominal Hydatidosis as Presumptive Sequelae of Primary Renal Echinococcosis: Clinical and Histopathologic Correlation

**DOI:** 10.7759/cureus.12968

**Published:** 2021-01-28

**Authors:** Demet Sengul, Ismail Aydin, Ilker Sengul, Tugrul Kesicioglu

**Affiliations:** 1 Pathology, Giresun University Faculty of Medicine, Giresun, TUR; 2 General Surgery, Giresun University Faculty of Medicine, Giresun, TUR; 3 Endocrine Surgery, General Surgery, Giresun University Faculty of Medicine, Giresun, TUR

**Keywords:** hydatidosis, hydatid cyst, echinococcosis, renal hydatid cyst, secondary abdominal hydatidosis

## Abstract

To present an extraordinary and likely first case of secondary multiple intraabdominal hydatidosis, probably as a sequel to primary renal hydatid cyst surgery. A 34-year-old male who underwent a right nephrectomy four years ago for renal hydatidosis was admitted. His abdominopelvic computed tomography scan revealed the hypodense lesions adjacent to the anterior lobe of the liver, splenic hilus, right common iliac vein, and left paracolic area. He underwent the total surgical resection and the histopathologic evaluation revealed the hydatidosis (echinococcosis) and the medical therapy, Albendazole: 15 mg/kg/day, was administrated for three months. No recurrence has occurred after the clinical and radiologic follow-up. To the best of our knowledge, it is an extreme and first case of secondary multiple intraabdominal hydatidosis as a sequel to the primary renal echinococcosis surgery, in the English language literature. Providers should be aware of considering a primary focus as encountering multiple intraabdominal hydatidosis.

## Introduction

Hydatid cyst is a zoonotic parasitic disease seen in humans and animals. The disease is prevalent in Australia, New Zealand, Middle East, Turkey, Greece, the Mediterranean, Latin America, and North Africa. It causes labor loss, complications, and even mortality. In addition to tapeworm *Echinococcus granulosus *(cystic echinococcus), some subtypes such as *E. multilocularis*, *E. oligarthrus,* and *E. vogeli*. are present in genus Echinococcus, family Taenidae, order Cyclophylidea, class Cestoda, phylum Platyhelminthes, and kingdom Animalia. However, *E. granulosus* is the causative agent in 95% of all cases [1,2].

It commonly affects the liver, 60-70%, and the lungs, 20-30%, while rarely affects the kidneys (2-3%) [3,4]. In the treatment, pericystectomy with partial or total nephrectomy is usually recommended [5]. Secondary abdominal hydatidosis can occur by a traumatic rupture of the parenchymal organs containing the hydatid cysts or in the form of an iatrogenic intraperitoneal spread. It may be isolated or multiple [6-8], and may also be located in the spleen, pancreas, omentum, pelvic abdomen, sigmoid colon, and retroperitoneum, intraabdominally [7-9]. It causes some non-specific clinical symptoms, such as abdominal pain, loss of appetite, and weight loss. It can also rarely lead to bleeding in the lower gastrointestinal system [10]. Diagnostic methods consist of abdominal computed tomography (CT) or ultrasonography (US), serologic test for Echinococcus immune hemagglutination (IHA), and histopathologic evaluation. A total surgical resection or possible external drainage is the treatment of choice [6]. The medical treatment, Albendazole and Praziquantel, is recommended to avoid secondary peritoneal echinococcosis [11].

## Case presentation

**Figure 1:**
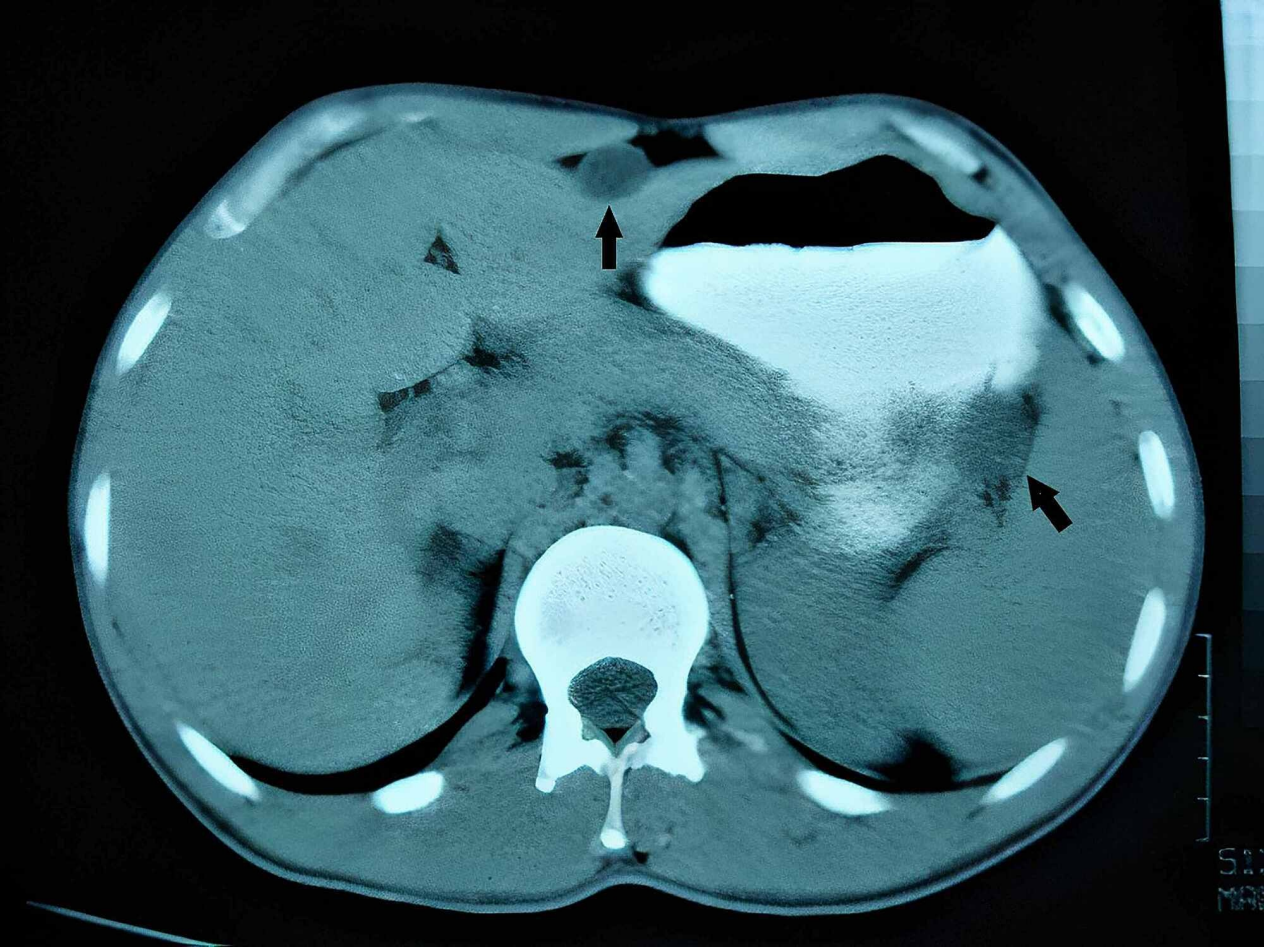
The radiologic image of hydatidosis in the vicinity of splenic hilus (arrow); abdomino-pelvic CT scan.

**Figure 2:**
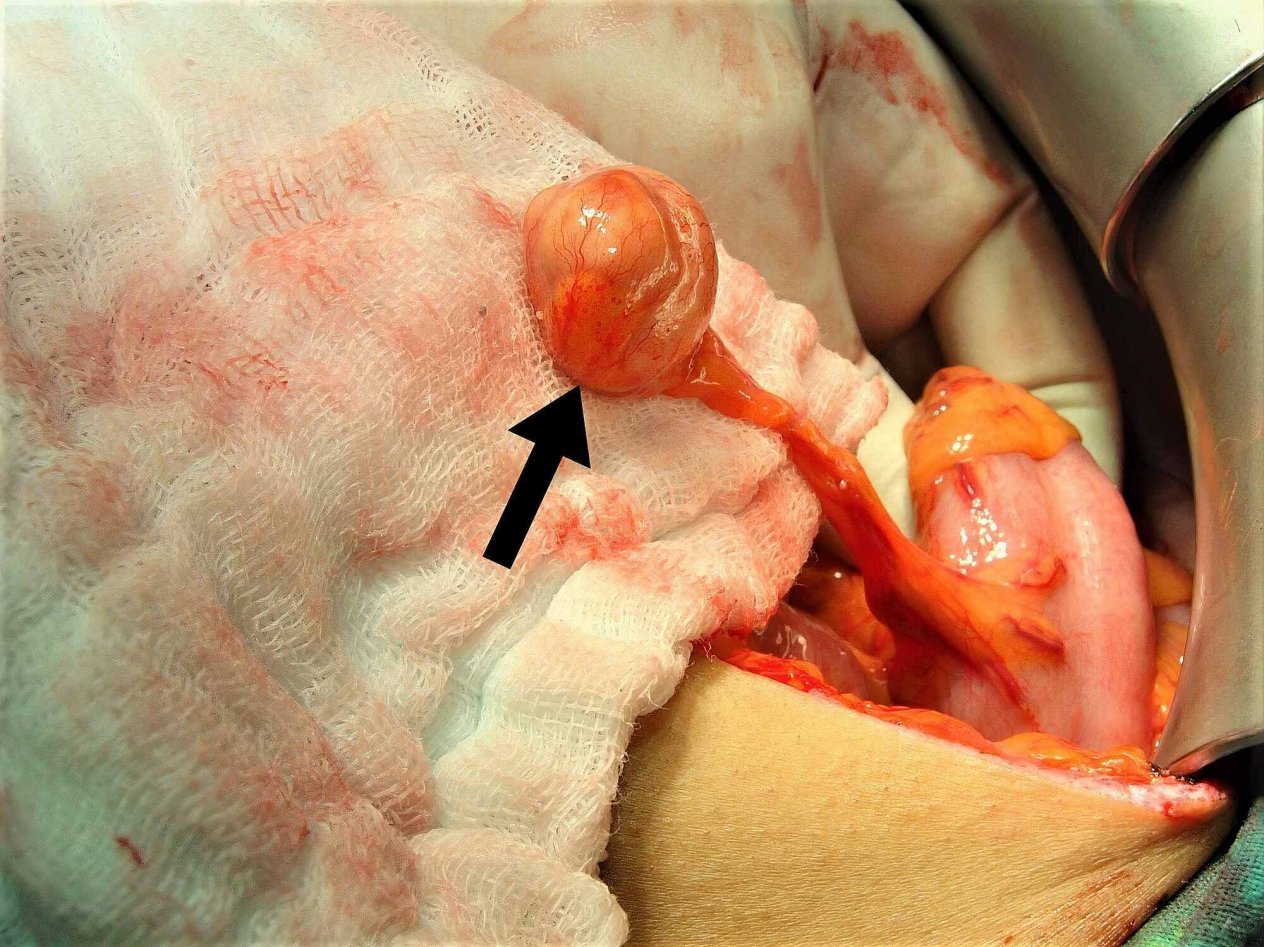
The intraoperative photograph, hydatid cyst on the sigmoid colon mesentery (arrow).

**Figure 3:**
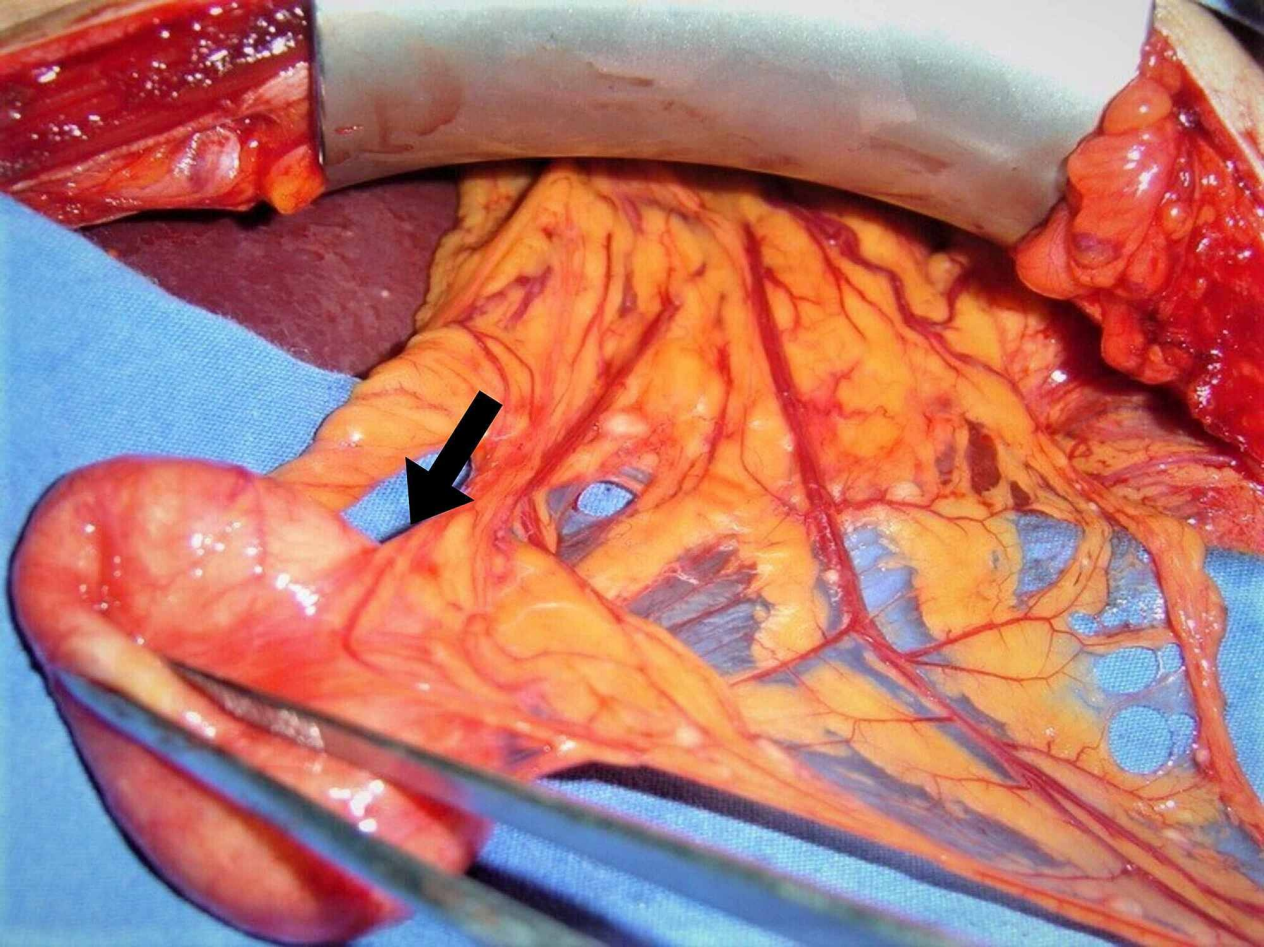
The intraoperative photograph, hydatid cyst in the vicinity of splenic hilus (arrow; shown as a radiologic image in Figure 1).

**Figure 4:**
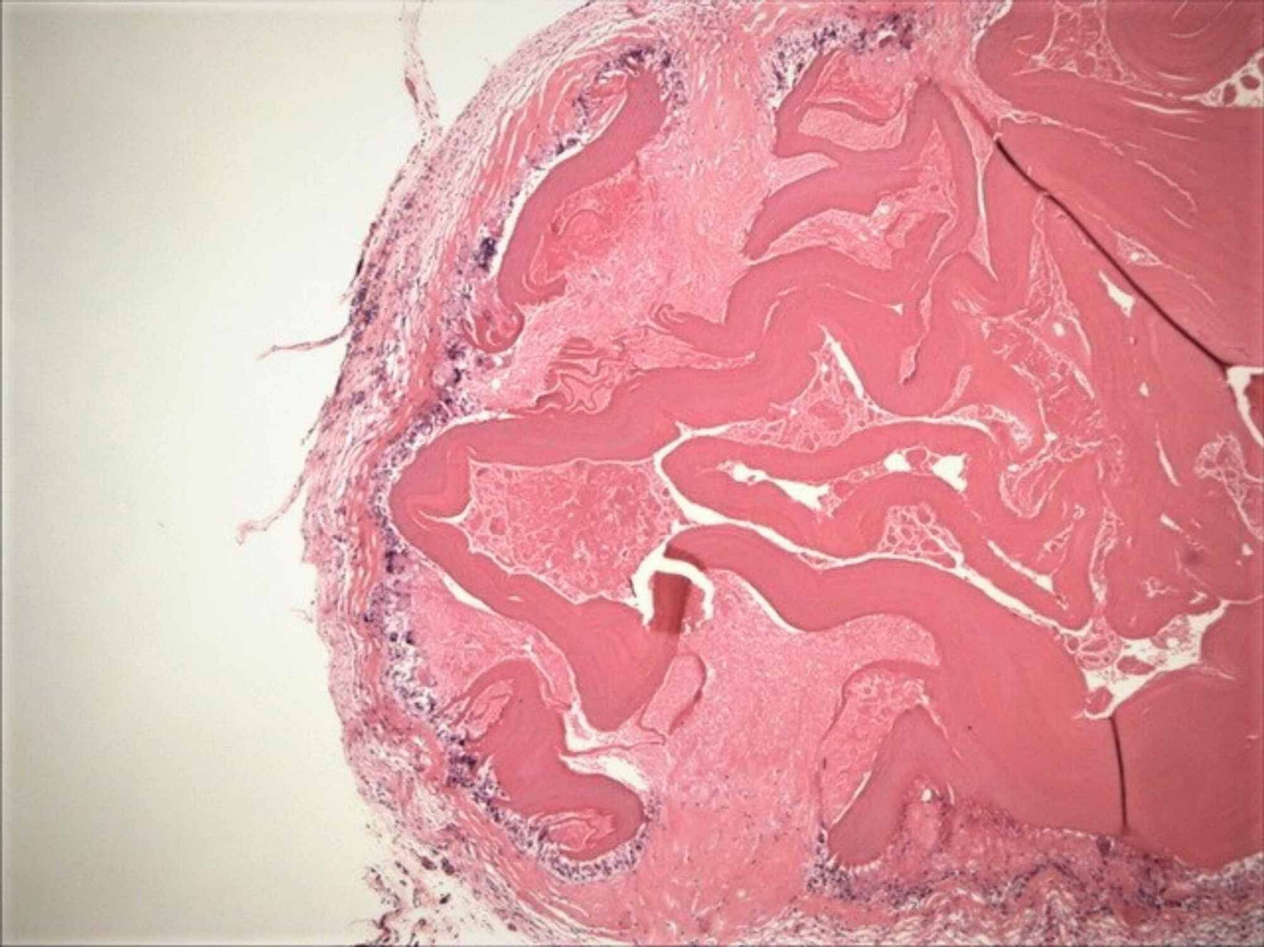
The photomicrograph of hydatidosis and their germinal membranes, on the falciform ligament (shown as a radiologic image in Figure 1; H&E; original magnification, 4 × 0.10). H&E: hematoxylin and eosin.

**Figure 5:**
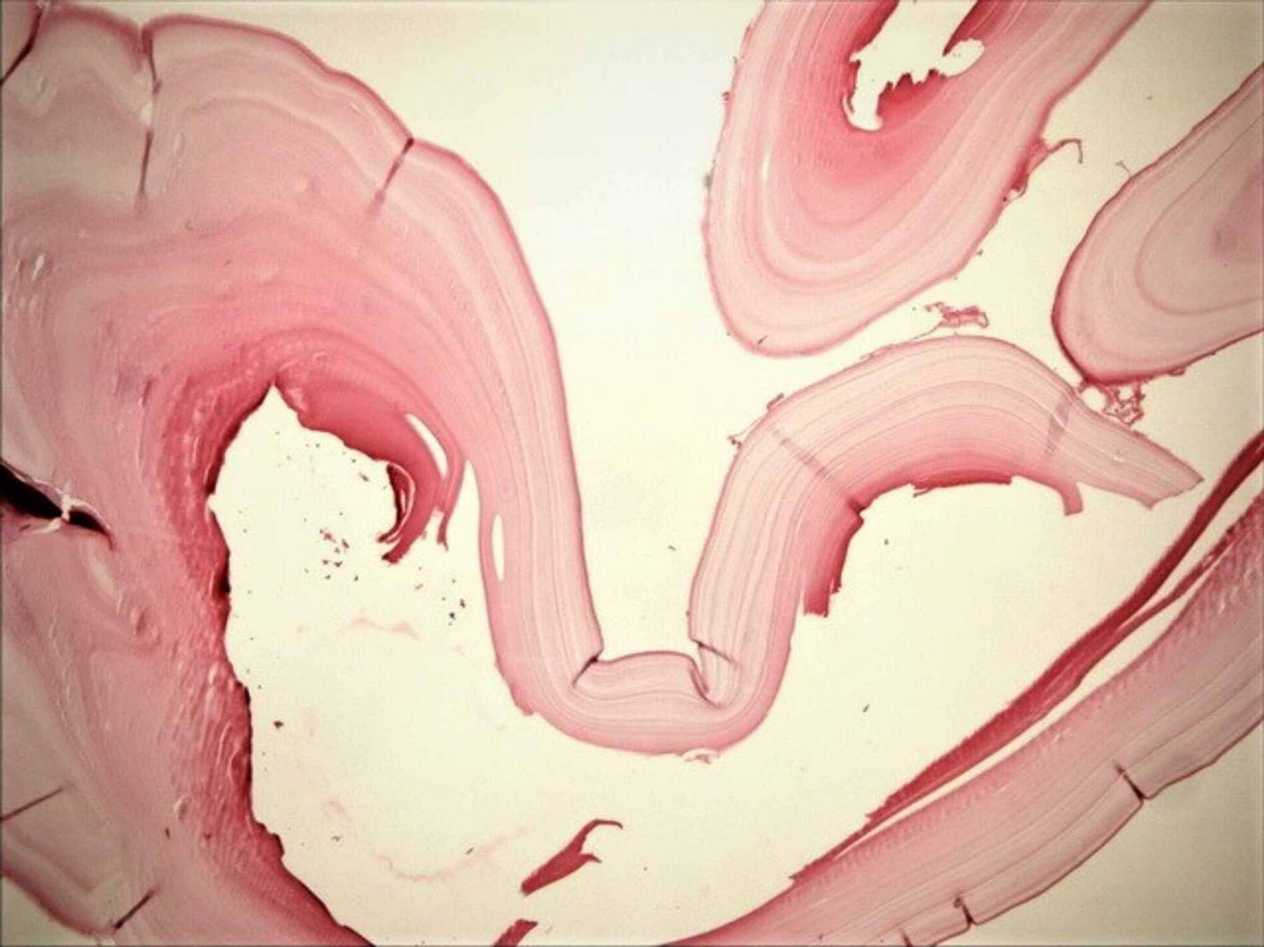
The photomicrograph of hydatidosis and their germinal membranes, in the vicinity of splenic hilus (shown as a radiologic image in Figure 1, intraoperative photograph in Figure 3; H&E: original magnification, 4 × 0.10). H&E: hematoxylin and eosin.

**Figure 6:**
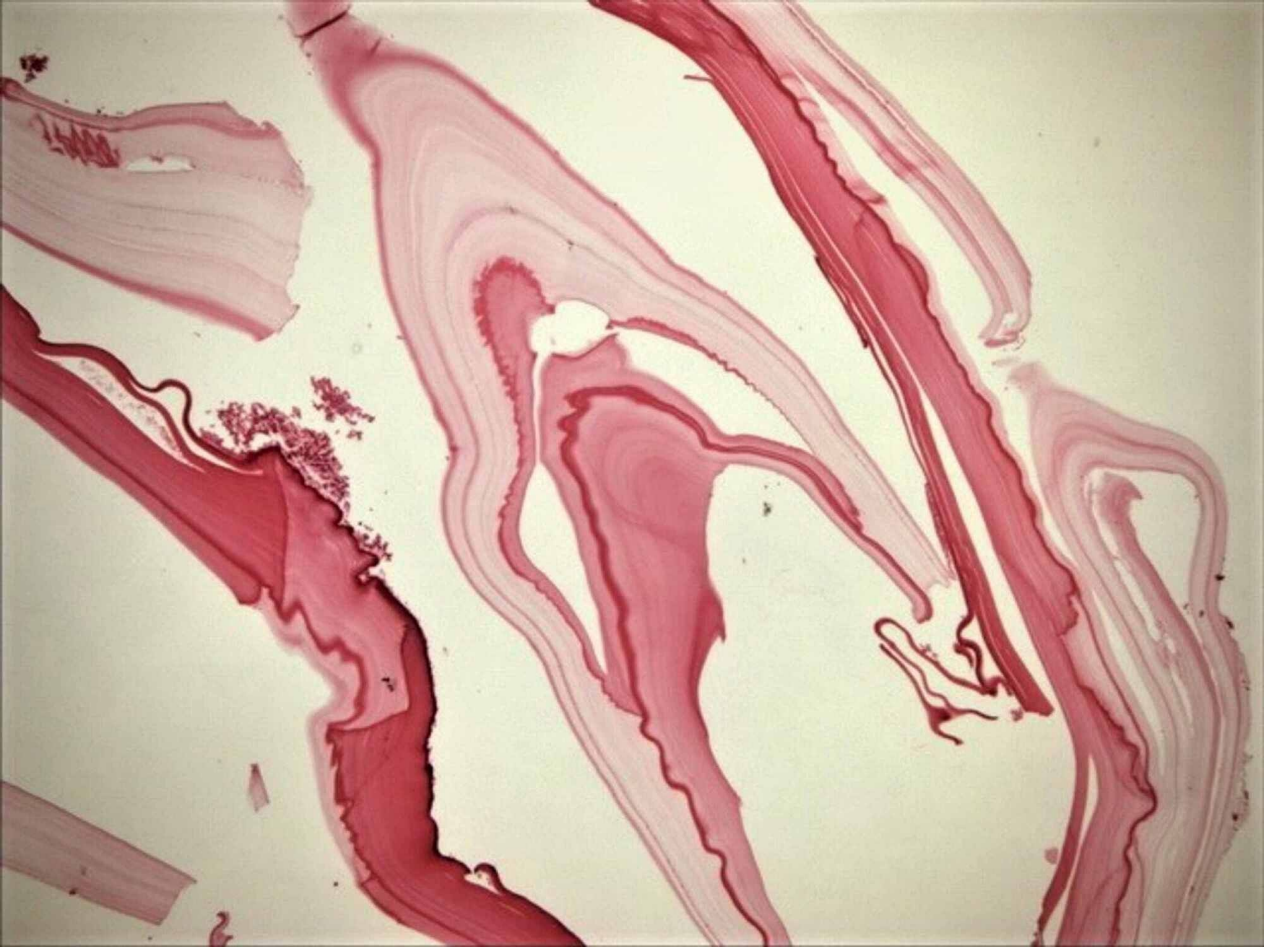
The photomicrograph of hydatidosis and their germinal membranes, on the momentum adjacent to the right paracolic area (H&E; original magnification, 4 x 0.10). H&E: hematoxylin and eosin.

**Figure 7:**
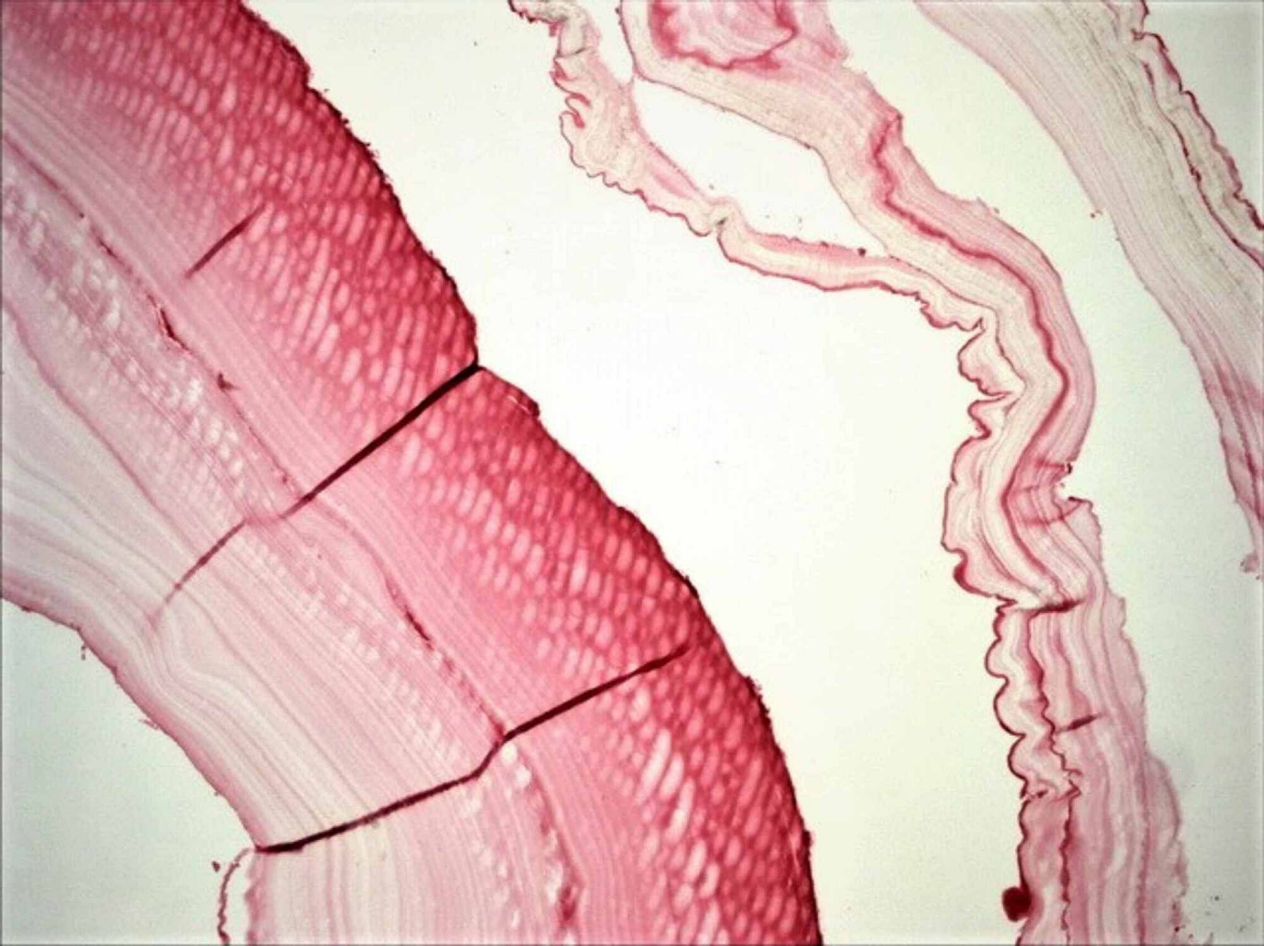
The photomicrograph of hydatidosis and their germinal membranes, on the sigmoid colon mesentery (H&E; original magnification, 4 × 0.10). H&E: hematoxylin and eosin.

A 29-year-old male was admitted with complaints of abdominal pain and loss of appetite for about six months. His personal medical history was unremarkable without any previous systemic diseases. On the physical examination, the epigastric, periumbilical, and suprapubic tenderness were recognized. He had a history of right nephrectomy due to the right renal hydatid cyst disease four years ago. The formal abdominopelvic CT scan hours after admission exhibited some hypodense lesions adjacent to the anterior lobe of the liver, splenic hilus, right common iliac vein, and right paracolic area, in the largest diameter of 15 × 5 mm^2^, 20 × 35 mm^2^, 30 × 20 mm^2^, and 45 × 15 mm^2^, respectively (Figure 1). The preoperative serologic test for Echinococcus IHA was positive. He was scheduled to undergo the surgical procedure with the preliminary diagnosis of secondary multiple intraabdominal hydatidosis. Before admission for surgery, there were no changes in clinical or biological status. The laparotomy was performed under general anesthesia using a midline supra- and partly infra umbilical incision after meticulous preparation. On the abdominal exploration, some cystic areas with a size of 1.5 cm on the falciform ligament adjacent to the anterior segment of the left lobe of the liver, multiple millimetric implants on the omentum majus, and 3 cm in the splenic hilus were recognized. In addition, 2 cm in the omentum adjacent to the right paracolic area, and 4 cm pedicle in the sigmoid colon mesentery were also manifested (Figures 2 and 3). All the described lesions were dissected and underwent a total surgical resection, meticulously. The histopathologic evaluation of the falciform ligament, splenic hilus, right paracolic area, and sigmoid mesentery revealed the echinococcosis (Figures 4-7). The postoperative period was uneventful and he was discharged on the fourth hospital day. The patient was prescribed to medical therapy, Albendazole: 15 mg/kg/day, for three months. No recurrence has occurred after the clinical and radiologic (US) follow-up to date [11].

## Discussion

A zoonotic parasitic disease of humans and mammalians, hydatidosis (hydatid disease) is the important parasitic disease of Australia, New Zealand, the Middle East, Turkey, Greece, Latin America, and North Africa bringing about some serious problems affecting public health and the national economy [1,2]. In hydatic disease, all organs can be involved, but mostly the liver is being affected worldwide [12]. The parasite can be placed primarily into the spleen, kidney, central nervous system, bone, heart, and muscle tissue [13,14]. Pain, mass, hydatidiform, hematuria, prolonged fever, and hypertension have been reported as the clinical features of this zoonotic disease, accounting for 63%, 26%, 11.4%, 31.4%, 23%, and 3%, respectively. Diagnostic methods consist of the serologic tests, IHA, abdominal CT, abdominal US, and histopathologic evaluation as usual [3]. Pericystectomy, partial nephrectomy, and total nephrectomy are the preferred methods for the treatment [5]. In the present case, the primary site of hydatid disease was the kidney, so it was intended to be treated with the nephrectomy, previously at another center.

Recurrence occurs in about 10% of cases treated for the hydatid disease and that is still a major problem in endemic regions such as our country, Turkey. In the literature, it has been reported and shown that the cyst dissemination into the abdomen, an inadequate cystic resection, and type and wideness of selected surgical method are important parameters in the disease recurrence [14].

If the cystic content can not be prevented from spreading during hydatid operation, it is seen that the disease has recurred years later, that is, secondary hydatidosis develops. For this reason, the killing of living scolexes in the cyst and carrying the proliferative potential are the most critical steps of the relevant operation [15]. Hydatid cyst fluid contains thousands of protoscoleces and endogenous capsules, which, when poured anywhere, can grow on the surface and lead to the development of mature hydatid cysts. Therefore, the ruptured hydatid cysts may fill the peritoneal cavity with multiple cysts and can lead to abdominal distension, intestinal obstruction, biliary peritonitis, biliary urticaria, angioneurotic edema, and anaphylaxis. One of the most important methods to be performed intraoperatively to avoid sequelae hydatidosis is the proper isolation of the surgical field. The cyst and cystic contents have to be completely isolated with surgical compresses impregnated with the scolicidal substance to kill the scoleces for avoiding their any possible already or further dissemination. Therefore, particular attention should be paid to ensure that the cyst content is not contaminated by intraperitoneal and surrounding tissues. Moreover, if peritoneal spread occurs perioperatively, it is recommended to wash the peritoneal cavity and scrape it with the relevant agents [16].

Secondary abdominal hydatidosis leads to non-specific clinical symptoms. In addition, as the cysts located in the peritoneal cavity, it may induce primary infertility as well as the symptoms of the digestive system and adjacent organs such as abdominal pain and general malaise. Besides, renal mass, hematuria, albuminuria, or hydatidosis can be seen in the kidney-located cysts [17]. In the present case, multiple secondary hydatid cysts in the abdomen, falciform ligament, spleen, right paracolic area, and sigmoid mesentery were exhibited to bring to mind the possibility of being an iatrogenic intraperitoneal spread at his previous surgical procedure. After a vigilant clinical evaluation and exhibition of suspicious imaging studies, the serologic laboratory tests should be performed before the histopathologic examination to reach the definitive diagnosis. The histopathologic evaluation reveals the cyst wall exhibiting an outer chitinous (or fibrous laminar) layer and an inner germinal layer. The cyst wall may be surrounded by a "pericyst layer": granulation tissue or a fibrose layer. If the latter has the calcifications, that indicates the relevant cyst is dead. However, the viable cysts are filled with colorless fluid, possessing daughter cysts and broad capsules with presences of scolices [3,6,18]. Surgical and external drainage treatments can be performed for secondary abdominal hydatidosis [6]. In our case, the multiple endocardial hydatid cysts were excised entirely by the open surgical procedure.

## Conclusions

In conclusion, secondary abdominal hydatidosis is a rare complication of hydatid disease. It can occur due to a rupture that could be spontaneous or more often following trauma and a perioperative iatrogenic peritoneal spread. Therefore, during the surgical treatment of a primary parenchymal organ's hydatid cysts, a meticulous surgery should be performed at each stage of the procedure to ensure that the cyst content will not meet the peritoneum, solid organs, and other vital structures. Once the secondary abdominal hydatidosis has been detected, it should be remembered that the definitive treatment is the total surgical resection with providing a meticulous exploration of the entire abdominal cavity to avoid the undesirable recurrence(s). The treatment by using Albendazole to prevent secondary peritoneal echinococcosis is recommended by all the authors, but there is no consensus regarding its duration. A minimum duration of three months is advised for medical treatment. Gastroenterologic and General Surgeons, Emergency Physicians, and also Pathologists should consider the possibility of being a sequel to primary renal hydatid cyst surgery or other relevant primary focus in secondary multiple intraabdominal hydatidoses.
